# Use of defaults on an electronic prescribing tool influences the type of fluid received by patients

**DOI:** 10.1186/cc12456

**Published:** 2013-03-19

**Authors:** L Herbert, C Bordeaux, M Thomas, L Burrows, J Bewley, T Gould

**Affiliations:** 1 Bristol Royal Infirmary, Bristol, UK

## Introduction

Many evidence-based interventions are not delivered to patients [1]. This may not be due to a clinician's intentional decisions. The aim of this project was to compare the use of starch before and after removing it as an option from an e-prescribing template.

## Methods

Our e-prescribing software enables users to prescribe intravenous fluids from a series of menus. One of these is a template that has several fluids available to use as a bolus when instructed by a clinician. We removed starch as an option from the template in April 2009. Starch could still be prescribed elsewhere on the prescribing system. Data on the use of starch from November 2008 to November 2012 were analysed as the mean volume of starch infused per patient per month. The mean of each set of parameters was then compared using a Student's *t *test.

## Results

**Figure 1:**
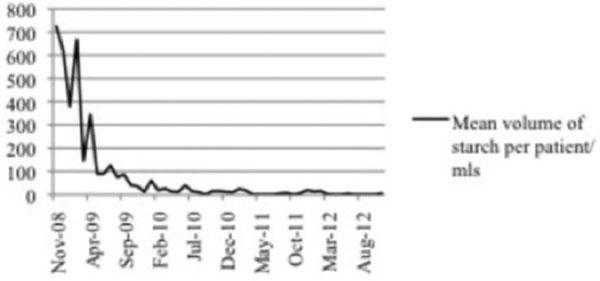
**Starch administration between November 2008 and November 2012**.

The mean volume of starch per patient administered before and after electronic prescription options were altered was 480 ml and 21 ml, respectively (*P *= 0.004). See Figure 1.

## Conclusion

Despite clinicians intending to reduce the use of starch it was still regularly administered on our ICU. The removal of a default prescribing option dramatically reduced the volume of starch used whilst not restricting the ability to make a conscious choice to prescribe it. Adjusting default options has potential to influence clinical decisions and ensure more reliable, evidence-based care.
